# Prevalence and Factors Associated with *Cryptosporidium* Infection in Calves in and around Nekemte Town, East Wollega Zone of Ethiopia

**DOI:** 10.1155/2022/1468242

**Published:** 2022-02-17

**Authors:** Adinew Ebiyo, Geremew Haile

**Affiliations:** ^1^Sibu Sire District Livestock Development Office, Nekemte, Ethiopia; ^2^School of Veterinary Medicine, Wollega University, Nekemte, Ethiopia

## Abstract

**Purpose:**

Cryptosporidiosis, caused by a protozoan parasite of the genus *Cryptosporidium*, is a zoonotic disease that affects young animals and humans. The study was conducted from October 2020 to May 2021, with the objectives of estimating the prevalence of *Cryptosporidium* infection in calves and assessing the associated risk factors in Nekemte town, Ethiopia.

**Methods:**

A cross-sectional study was adopted during the study period. Initially, 35 dairy farms were selected using a systematic study approach from which a total of 384 calves under the age of 12 months (75 calves from intensive, 51 from semi-intensive, and 258 from extensive dairy farms) were selected purposively for fecal sample collection. Fecal samples were collected directly from the rectum of each calf and tested for *Cryptosporidium* oocysts using Sheather's flotation and a modified Ziehl–Neelsen staining procedure. The data were analyzed using STATA statistical software version 13.

**Results:**

*Cryptosporidium* infection was found in 53 of the 384 calves investigated, with an overall prevalence of 13.8% (95% CI = 8.1–17.6). Logistic regression analysis on the risk factors showed that types of farm, age, body condition scores, fecal consistency, types of feed, source of water, and farm hygiene were found to have a statistically significant influence on the shedding of *Cryptosporidium* oocysts by the calves (*p* < 0.05). Calves under the age of six months had a higher likelihood (OR = 2.7, 95% CI = 1.0–4.2, *p* < 0.001) of shedding the oocysts than the calves of 6 to 12 months of age. It was also confirmed that calves with poor body condition scores had a higher likelihood (OR = 2.6, 95% CI = 1.2–3.7, *p*=0.024) of shedding the *Cryptosporidium* oocysts than other ones. The likelihood of shedding a *Cryptosporidium* oocyst by the diarrheic calves was higher than by the nondiarrheic ones (OR = 3.1, *p* < 0.001). The infection was 2.6 times highest (16.8%, 31/185, 95% CI = 14.5–19.1, OR = 2.6, *p*=0.028) in calves feeding on grass alone, followed by milk and grass (15%) and milk (4.2%). Similarly, it was 2.2 times higher (18.5%, OR = 2.2, and *p*=0.002) in calves drinking river water than in calves drinking tap water. Moreover, calves kept under poor hygiene were more likely to shed *Cryptosporidium* oocysts (OR = 2.8, *p*=0.001) than calves kept under good hygiene.

**Conclusion:**

In conclusion, cryptosporidiosis is one of the health problems of calves in and around Nekemte town, East Wollega zone. Our findings confirmed that risk factors such as types of farm, age, body condition scores, fecal consistency, types of feed, water source, and farm hygienic status were found to have a significant effect on the occurrence of *Cryptosporidium* infection in the calves. While the molecular-based study is required to be carried out aiming at species identification and the epidemiology of the parasite, particular attention should be paid to the control of the disease in the study area.

## 1. Introduction

Ethiopia has the largest livestock population in Africa with an estimated cattle population of 53.9 million, of which female cattle constitute about 55.4%. Livestock provides draft power, income to farming communities, investment opportunities, and a source of foreign exchange to the country [1–3]. Cattle productivity is mostly determined by their reproductive ability and calves' survival. Calf morbidity and mortality are significant problems for dairy farmers all over the world [4]. Infectious diseases are the leading causes of calf deaths. Diarrhea is the most common symptom of sickness in young calves, and it accounts for about 75% of calves' deaths in the first three weeks of life [5, 6]. Among the most common causes of diarrhea in calves are rotavirus, *Escherichia coli*, *Salmonella*, *Cryptosporidium*, and *Giardia* [7, 8].

Cryptosporidiosis, caused by a protozoan parasite of the genus *Cryptosporidium*, is a zoonotic disease that causes illness and significant losses in calves and young cattle. *Cryptosporidium* can develop and multiply in the gastrointestinal tracts which causes diarrhea in the infected host [9–11]. The economic impact of the disease is attributed to high morbidity, high mortality, low performance, and high cost of treatment [12]. The disease has significant public health concern [13, 14]. Cattle are considered potentially important reservoirs for human infections [15]. Infections are commonly transmitted by ingestion, inhalation of oocysts, and collateral contact with infective stages of the fully sporulated oocysts when excreted [16, 17].

Cryptosporidiosis is distributed worldwide, infecting a wide range of hosts; however, the disease is most commonly diagnosed in cattle [18], and about 24 species and over 40 genotypes of *Cryptosporidium* are detected with four primary species, *Cryptosporidium parvum*, *C. andersoni*, *C. ryanae*, and *C. bovis*, being the most common. Other species have been found in cattle, such as *C. suis*, *C. hominis*, *C. xiaoi, C. ubiquitum*, *C. meleagridis*, *C. muris*, and *C. felis* [19]. Molecular-based studies have indicated that at least 15 different species are involved in human sickness, with *C. hominis*, *C. meleagridis*, *C. felis*, and *C. canis* being common [20]. Host-related characteristics such as age, sex, and breed, as well as environmental factors like housing, are the major risk factors for cryptosporidiosis in calves [21, 22]. Hygienic status, feed, and water sources are other important factors [23, 24].

A variety of methods are available for the detection of *Cryptosporidium* including microscopic, immunological, and molecular techniques. Microscopic detection is based on finding the oocysts in fecal samples. Oocysts may be demonstrated using by Ziehl–Nielsen stained fecal smears in which the sporozoites appear as bright red granules [7, 21]. The design of a strategic plan to control and prevent cryptosporidiosis depends largely on understanding the factors that lead to the introduction, transmission, and spread of infection in animals and humans.

Research has been done on the prevalence of bovine cryptosporidiosis in different parts of Ethiopia [25–29]. However, no research has been performed in the East Wollega zone, where there are commonly filed cases of diarrheic calves. This research will be critical for developing long-term disease management strategies in the future. Therefore, the overall objectives of this study were to estimate the prevalence of *Cryptosporidium* infection in calves and to assess the potential risk factors associated with the disease in and around Nekemte town.

## 2. Materials and Methods

### 2.1. Study Area

**Figure 1:**
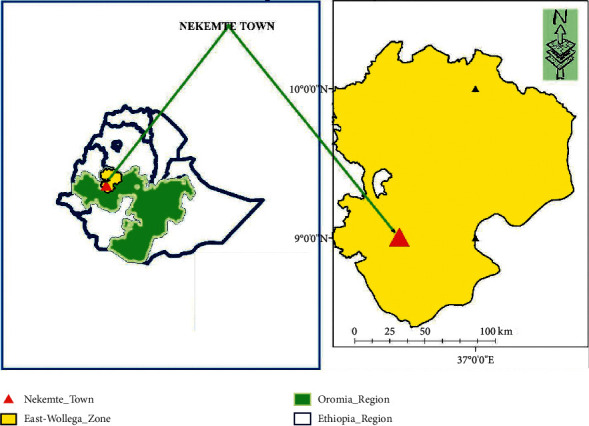
Map of the study area, Nekemte town, East Wollega zone, Ethiopia.

The study was conducted in and around Nekemte town, East Wollega zone, western part of Oromia, Ethiopia (Figure 1). Nekemte is located about 328 kilometers from Addis Ababa. The East Wollega zone of the Oromia Region is administratively divided into 21 woredas. The area is agro-ecologically split into 11% highlands, 49% midlands, and 40% lowlands. The zone has one long rainy season extending from March to mid-October, with annual rainfall ranging from 1000 mm. Mixed agriculture (crops and livestock) is the mainstay of the farming communities on which their livelihood is fully dependent [30, 31].

### 2.2. Study Population

All of the study animals were calves under the age of 12 months and were maintained on intensive, semi-intensive, and extensive dairy farms in and around Nekemte town. Calves of all breeds (local, exotic, and crossbreeds) and both sexes were included in the study. Body condition scores were recorded as poor, medium, and good by observing the body condition of the calf [32]. The calves' age was determined based on dentation [33], owners' responses, and written records.

### 2.3. Study Design

Between October 2020 and May 2021, a cross-sectional study design was used to estimate the prevalence of *Cryptosporidium* infection in calves and assess associated risk factors.

### 2.4. Sample Size Determination

Because there had been no previous study published on the prevalence of *Cryptosporidium* infection in the study area, a total of 384 calves were included based on the formula given by Thrusfield [34] at 50% expected prevalence with 5% precision and a 95% confidence range.

(1) $$\begin{array}{c} n=\frac{{\left(1.96\right)}^{2}p\;\exp\left(1-p\;\exp\right)}{d{\;}^{2}}=\frac{{\left(1.96\right)}^{2}\;0.5\left(1-0.5\right)}{{\left(0.5\right)}^{2}}, \end{array}$$

where *n* = sample size, *p*exp = expected prevalence, and *d*^2^ = desired absolute precision.

### 2.5. Sampling Technique

A systematic random sampling technique was used to include 35 dairy farms (6 intensive, 10 semi-intensive, and 19 extensive dairy farms) from the study area. The study calves were chosen purposively from the dairy farms depending on the number of calves available on the selected farms.

### 2.6. Study Methods

Fecal samples were collected directly from the rectum of each calf by using disposable gloves and placed in a universal bottle and labeled. At the time of sample collection, the sampling date, calf's age, sex, breed, body condition score, fecal consistency (normal/diarrhea), feed, and drinking water source were recorded for each animal on a recording sheet. The collected fecal samples were preserved in 10% formalin and transported in an icebox to the Veterinary Parasitology Laboratory of Wollega University for laboratory analysis. The samples were then processed by fecal flotation using Sheather's sugar solution. Fecal smears were also prepared and stained using a modified Ziehl–Neelsen acid-fast stain. The slides were examined microscopically at 100x objective using oil immersion. The samples were recognized as positive for the *Cryptosporidium* oocysts based on the oocyst color, which appeared as bright red granules on a blue background [35, 36].

### 2.7. Data Analysis

All the collected data were managed in a Microsoft Excel spreadsheet. STATA software version 13 was used for data analysis. The effect of the different risk factors associated with the infection was observed using binomial and multivariate logistic regression, and the association between *Cryptosporidium* infection and risk factors was said to be significant when *pp* value<0.05. The overall prevalence was computed by dividing the number of *Cryptosporidium*-positive animals by the total number of animals examined and multiplying it by 100.

## 3. Results

Of the 384 examined calves, *Cryptosporidium* oocysts were recorded in 53 calves with an overall prevalence of 13.8% (95% CI = 8.1–17.6). The occurrence of *Cryptosporidium* oocysts among the types of dairy farms was compared using the Chi-square test, and a significant difference of the *Cryptosporidium* occurrence was observed among the dairy farms (*X*^2^ = 10.4, *p*=0.006) (Table 1).

Table 2 shows the logistic regression analysis of risk factors associated with *Cryptosporidium* infection. Accordingly, a higher infection was recorded in local breed calves (14.5%, 44/303) followed by cross (11.8%, 2/17) and exotic breed (10.9%). Female calves had a higher prevalence (15.3%) than males (11.4%). A statistically significant difference was not observed between the occurrence of the *Cryptosporidium* oocyst and the breeds and sex of the calves. Shedding of *Cryptosporidium* by calves significantly varied with age groups and body condition scores. Calves under six months of age had a higher likelihood (OR = 2.7, 95% CI = 1.0–4.2, *p* < 0.001) of shedding the oocysts compared to calves of 6 to 12 months of age. Similarly, calves with poor body condition scores had a higher likelihood (OR = 2.6, 95% CI = 1.2–3.7, *p*=0.001) of shedding the oocysts compared to other ones (Table 2).

Logistic regression analysis on the risk factors such as fecal consistency, feed types, sources of water, and farm hygiene was also computed (Table 3). Accordingly, the shedding of *Cryptosporidium* oocysts by the diarrheic calves was significantly higher than that of the nondiarrheic ones. It was also confirmed that the shedding of oocysts was high (16.8%, 31/185, 95% CI = 15.9–22.8, OR = 2.6) in calves feeding on grass alone, followed by milk and grass (15%) and milk (4.2%). Similarly, water sources significantly affected the infection rate, indicating higher infection (18.5%, OR = 2.2, *p*=0.002) in calves drinking river water when compared with calves drinking tap water. Moreover, farm hygiene status also significantly affected the infection. It was three times more in calves kept under poor hygiene (OR = 2.8, *p*=0.001) than in those calves kept under good hygiene.

## 4. Discussion

The overall prevalence of *Cryptosporidium* infection in calves was 13.8% in this investigation. Our findings are consistent with a study conducted in Bishoftu, Ethiopia, which found a total prevalence of *Cryptosporidium* infection of 13.6% in calves [26]. It also matched the findings of studies undertaken in Norway [37] and India [38], which found prevalence rates of 11.7% and 12%, respectively. The current finding, on the other hand, was lower than that of Regassa et al. [25], who documented 27.8% in dairy calves in Haramaya, East Hararghe, Ethiopia. It was also lower than the findings from Johor (27.1%) [39], Vietnam (33.5%) [40], and Malaysia (35%) [41]. However, the present finding was relatively higher than that of Ayele et al. [29], who reported 9.4% *Cryptosporidium* infection in dairy calves in Central Ethiopia. It is also higher than the reports in different parts of the world, which range from 2.66% to 7.5% in China [42], Turkey [43], and Japan [44]. These differences in the *Cryptosporidium* infection among the countries might be attributed to the differences in livestock production systems, agro-ecological differences, sample size, and laboratory techniques. According to Gowda et al. [10], the diagnostic techniques employed in the present survey are less sensitive and can give false-negative results.

The Chi-square tests on the types of dairy farms showed that the occurrence of the *Cryptosporidium* infection significantly varied between the dairy farms. More infections were recorded in small-scale dairy calves than in other ones (*X*^2^ = 10.4, *p*=0.006). This difference might be attributed to a difference in the sample size and management practice. The present study showed that the occurrence of the *Cryptosporidium* infection was significantly associated with the age of the calf, indicating the calves under six months of age had a higher risk of infection as compared to the older ones (OR = 2.7, *p* < 0.05). This finding is in line with the previous reports in [25, 26, 28], which reported the significant association of *Cryptosporidium* infection with age. This might be because of the immature immune system of young calves. This is supported by a study [24] which described that resistance to infection could be developed with age due to immune development through time.

It has also been observed that the occurrence of *Cryptosporidium* oocysts was strongly associated with body condition scores of calves, which our results confirmed, the highest infection being in calves with poor body condition scores (OR = 2.6, *p* < 0.05). This is in agreement with the report of Nasir et al. [24]. The highest infection rate in calves with poor body condition might be due to the fact that the parasite can cause chronic infections or might be attributed to the poor management system of the calves, which results in susceptibility to *Cryptosporidium* infections. A substantial relationship was also found between the prevalence of *Cryptosporidium* infection and fecal consistency, with diarrheic calves shedding the oocysts more frequently than those with normal fecal consistency, which agrees with a study by Wegayehu et al. [28]. This could be due to the fact that *Cryptosporidium* is an enteric parasite that causes the loss of epithelial cells and microvillus of the intestine as well as a reduction in the absorptive surface area of the intestine, resulting in diarrhea [19].

A strong association was observed between the types of feed and the occurrence of *Cryptosporidium* infection in which the free-grazing calf was more infected than the calf on other feed sources. This is in line with studies by Nasir et al. [24], who reported a strong association between types of feed and *Cryptosporidium* infection. A significant association was also found between the water source and *Cryptosporidium* infection, with a higher occurrence of the infection in calves given river water than in those calves given tap water, which is in agreement with the study in [6, 17]. The strong association between the types of feed and water sources might be attributed to the contamination of grass and water with *Cryptosporidium* oocysts and with the feces of humans and domestic and wild animals [7].

A significant association was also observed between farm hygiene and the occurrence of *Cryptosporidium* infection in calves. The present report is supported by the findings in [27, 29] which described the significant association of the *Cryptosporidium* infection with the poor hygienic status of the farm. This could be explained by the fact that dirty and muddy husbandry could create favorable microclimatic conditions for the survival of *Cryptosporidium* oocysts in animal houses, which leads to an increase in feed and water contamination.

## 5. Conclusion and Recommendations

The present study concluded that cryptosporidiosis is one of the health problems of calves in the study area, with an overall *Cryptosporidium* infection occurrence of 13.8%. Among the risk factors included in this study, types of farm, age, body condition scores, hygienic status, feed, and water sources were found to be the potential risk factors for the occurrence of the pathogen. Therefore, effective control strategies such as good farm hygiene should be implemented. Community awareness creation regarding the public health and economic significance of cryptosporidiosis should be practiced in the study area. Moreover, further studies should be carried out on species identification and molecular characterization aimed at understanding the epidemiology of the disease.

## Figures and Tables

**Table 1:** Prevalence of cryptosporidiosis in calves based on the types of the farms.

Types of dairy farms	No. of calves examined	No. of positive samples	Prevalence (95% CI)	*X* ^2^	*p* value
Intensive	75	4	5.3 (3.1–9.4)	10.4	0.006
Semi-intensive	51	8	15.7 (8.1–17.9)		
Small-scale	258	36	13.9 (9.6–18.4)		
**Overall prevalence**	**384**	**53**	**13.8 (8–17.6)**		

**Table 2:** Logistic regression analysis of risk factors associated with *Cryptosporidium* infection.

Risk factors	No. of calves examined	No. of positive samples	Prevalence (95% CI)	OR (95% CI)	*p* value
Breed					
Exotic	64	7	10.9 (4.3–17.2)	1	
Cross	17	2	11.8 (6.2–16.4)	1.04 (0.2–1.8)	0.729
Local	303	44	14.5 (9.6–20.3)	1.3 (0.67–2.3)	0.517
Sex					
Male	149	17	11.4 (7.1–18.4)	1	
Female	235	36	15.3 (9.8–21.9)	1.4 (0.8–2.4)	0.279
Age					
6–12 months	293	30	10.2 (7.5–20.6)	1	
<6 months	91	23	25.3 (15.8–29.2)	2.7 (1.0–4.2)	<0.001
Body condition scores					
Good	116	9	7.8 (4.9–12.1)	1	
Medium	207	26	12.6 (7.7–15.4)	2.4 (0.99–3.6)	0.024
Poor	61	18	29.5 (18.3–36.3)	2.6 (1.2–3.7)	0.001
**Overall prevalence**	**384**	**53**	**13.8 (8.1–17.6)**		

**Table 3:** Logistic regression analysis of fecal consistency and calf management with *Cryptosporidium* infection.

Risk factors	No. of calves examined	No. of positive samples	Prevalence (95% CI)	OR (95% CI)	*p* value
Fecal consistency					
Normal	311	32	10.3 (7–13.5)	1	
Diarrhea	73	2 1	28.8 (22.1–33.6)	3.1 (1.6–5.1)	<0.001
Types of feed					
Milk	72	3	4.2 (2.2–6.7)	1	
Milk and grass	127	19	15 (11–17.3)	2.2 (1.2–4.4)	0.037
Grass	185	31	16.8 (14.5–19.1)	2.6 (1.3–4.8)	0.028
Water sources					
Tap water	168	13	7.7 (6.7–12.9)	1	
River	216	40	18.5 (14.5–20.2)	2.2 (1.1–3.9)	0.002
Farm hygiene					
Good	180	14	7.8 (5.4–12.7)	1	
Poor	204	39	19.1 (15.6–22.5)	2.8 (1.4–5.1)	0.001
**Total**	**384**	**53**	**13.8 (8.1–17.6)**		

## Data Availability

The data used to support the findings of this study are available and will be included upon request to the corresponding author.

## Additional Points

The parasitological techniques used for laboratory analysis were insufficiently sensitive to allow for accurate parasite identification. As a result, molecular-based research will be required in the future.
